# Fibroblast miR-210 overexpression is independently associated with clinical failure in Prostate Cancer – a multicenter (*in situ* hybridization) study

**DOI:** 10.1038/srep36573

**Published:** 2016-11-08

**Authors:** Sigve Andersen, Elin Richardsen, Line Moi, Tom Donnem, Yngve Nordby, Nora Ness, Marte Eilertsen Holman, Roy M. Bremnes, Lill-Tove Busund

**Affiliations:** 1 Translational Cancer Research Group, Dept Clinical Medicine, UiT, The Arctic University of Norway, 9037 Tromso, Norway; 2 Dept Oncology, University Hospital of North Norway, 9038 Tromso, Norway; 3 Translational Cancer Research Group, Dept of Medical Biology, UiT, The Arctic University of Norway, 9037 Tromso, Norway; 4 Dept Pathology, University Hospital of North Norway, 9038 Tromso, Norway; 5 Dept of Urology, University Hospital of North Norway, 9038 Tromso, Norway.

## Abstract

There is a need for better prognostication in prostate cancer (PC). “The micromanager of hypoxia”, microRNA-210 (miR-210) is directly linked to hypoxia, is overexpressed in PC and has been implied in tumor cell-fibroblast crosstalk. We investigated the prognostic impact of miR-210 in tumor cells and fibroblasts in PC. Tumor and stromal samples from a multicenter PC cohort of 535 prostatectomy patients were inserted into tissue microarrays. To investigate the expression of miR-210, we used *in situ* hybridization and two pathologists semiquantitatively scored its expression. Overexpression of miR-210 in tumor cells was not associated to biochemical failure-free survival (BFFS, p = 0.85) or clinical failure-free survival (CFFS, p = 0.09). However, overexpression of miR-210 in fibroblasts was significantly associated to a poor CFFS (p = 0.001), but not BFFS (p = 0.232). This feature was validated in both cohorts. Overexpression of miR-210 was independently associated with a reduced CFFS (HR = 2.76, CI 95% 1.25–6.09, p = 0.012). Overexpression of miR-210 in fibroblasts is independently associated with a poor CFFS. This highlights the importance of fibroblasts and cellular compartment crosstalk in PC. miR-210 is a candidate prognostic marker and potential therapeutic target in PC.

There is an unacceptable situation in prostate cancer (PC) with considerable overtreatment leading to unnecessary side effects^1^. On the other hand, PC is a major cancer killer^2^. Current diagnostics, screening and treatment optimization are not able to overcome this problem^3^. A way forward might be identification and validation of biomarkers with significant prognostic or predictive capabilities with the composite pre-biopsy STHLM3 model as a promising example^4^.

In early cancer research, most researchers focused on the cancer cells alone. However, interactions between neoplastic cells (tumor) and surrounding stromal cells (stroma) are increasingly being accepted as important for cancer dynamics^5^. Stromal cells, as lymphocytes, macrophages and fibroblasts, are interspersed in the tumor compartments and enclose PC tumor cells. Cancer-associated fibroblasts (CAFs) are the dominant stromal cell in PC. CAFs have been shown to influence both invasion and metastasis^6^ and have been discussed as targets for theraphy^7^.

Hypoxia is a common feature in solid tumors and is known to upregulate tumor development, growth and metastasis in general^8^, including prostate cancer^9^, and to drive prostatic tumor-stroma co-evolution^10^. The hypoxia-inducible factors (HIFs), with the emphasis on the oxygen sensitive subunit HIF-1α, are recognised as being the major players of the hypoxia signalling pathways^11^. HIF1α specifically regulates cellular adaption to hypoxia through microRNAs (miRs)^12^. miRs are small, single stranded noncoding RNAs which regulate protein expression by interference with mRNA. A single miR can affect translation of >100 mRNAs and at least 60% of exons are under control by a miR^13^, illustrating their potential influence in cell function.

miR-210 has been appointed “the micromanager of hypoxia” as it is the most responsive and influential miR regulated by the HIF pathways^12^. It is also a marker of hypoxia as miR-210 stabilizes HIF1α which in turn increases the expression of miR-210^12^. In cancer, most studies have found miR-210 to be associated with poor prognosis, (reviewed in^14^,^15^,^16^). In PC specifically, miR-210 has been found overexpressed in tumor tissue^17^, in blood of metastatic patients^17^,^18^. In serum, miR-210 has been found to correlate to treatment response in metastatic prostate cancer. Although its importance in cancer progression has been stated^19^, there are conflicting reports regarding its clinical usefulness as a prognostic marker. As PC is a multifocal tumor, an *in-situ* specific approach might be better suited to unravel function and prognostic impact. However, no study has investigated the prognostic role of miR-210 in PC, neither by regular expression data nor with an *in-situ* approach for tissue differentiation.

We have previously explored the expression and prognostic impact of miR-210 in NSCLC^20^. In this study, we aimed to assess the expression of miR-210 in both fibroblasts and tumor cell PC tissue by chromogenic *in-situ* hybridization (ISH). But more important, by using our recently updated, well described and largely unselected PC cohort^21^, we were able to perform an investigation of the potential prognostic impact of miR-210.

## Results

### Expression of miR-210

**Figure 1:**
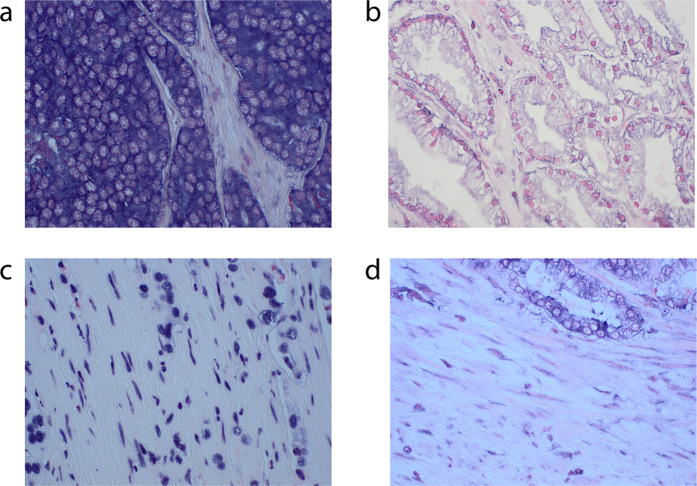
400× microscope pictures of (**a**) high tumor cell miR-210 expression, (**b**) Low tumor cell miR-210 expression, (**c**) high fibroblast miR-210 expression, (**d**) low fibroblast miR-210 expression.

Photographs showing low and high expression examples of tumor cell (panel A) and fibroblast miR-210 (panel B) in PC are presented in Fig. 1. Interobserver scoring agreement for scoring miR-210 in tumor cells were: ICC = 0.79, p < 0.001; for miR-210 fibroblast intensity: ICC = 0.80, p < 0.001 and for fibroblast density: ICC = 0.85, p < 0.001. There was specific cytoplasmic staining with a slightly more accentuated intensity close to the cell membrane. Lymphocytes, macrophages and smooth muscle cells were also stained when present.

Of the total cohort, 345 patients had cores with morphologically verified malignant cells available for scoring. 190 patients either had all cores missing or no morphologically verified malignant cells present in the cores. For fibroblasts, only 38 patients had no scorable tissue. In tumor cells the mean expression score was 2.26, (range 1–3) and the most prevalent score was 3 (28.4%). For the summarised expression of intensity and density in fibroblasts the mean expression score was 5.39 (range 1.5–6) and the most prevalent score was 6 (38.4%). There were no tumor or fibroblast cores without miR-210 expression.

### Correlations

There was a significantly positive correlation between tumor cell and fibroblast miR-210 expression (*r* = 0.32; p < 0.0001). As we have expression data on many previously published markers in this cohort, we did a broad inclusion of the markers that to our knowledge could be associated to endothelial mesenchymal transition (EMT), angiogenesis, aerobic glycolysis and immunological adaptations. These pathways were by Taddei *et al*. found to be induced in an environment with increased miR-210 expression^22^. We also correlated expression to all clinicopathological variables in this cohort as these variables were included due to their possible association to prognosis (see Table 1). We found tumor cell expression of miR-210 to correlate significantly to; VEGFR-2 in tumor cells (*r* = 0.185, p = 0.001) and the clinicopathological variables vascular infiltration (*r* = 0.173, p = 0.001), perineural infiltration (*r* = 0.109, p = 0.042) and pTstage (r = 0.166, p = 0.002). miR-210 expression in fibroblasts, correlated to VEGFR-2 expression in stroma (*r* = 0.171, p < 0.001) and CD3+ cells in stroma (*r* = 0.172, p = 0.024).

### Univariate analyses

**Figure 2:**
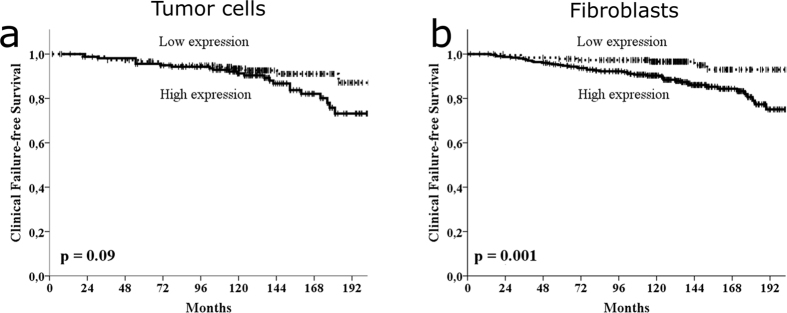
Kaplan-Meier curves, illustrating clinical failure-free survival for the whole cohort for low and high expression of miR-210 in (**a**) tumor cells and (**b**) fibroblasts. The p-value is the univariate log rank p-value.

For tumor cell miR-210 expression, no statistical significant cut-off was associated with BFFS or CFFS. However, a trend between high expression and worse CFFS was present (p = 0.090, Table 1 and Fig. 2a). For fibroblast miR-210 expression, there was no significant association to BFFS for the mean cut-off (p = 0.23, Table 1). However, the trend was always towards worse BFFS for all cut-offs and there was a significant association to a worse BFFS for the optimal cut-off of 4.5 (p = 0.034). Regarding the clinically more relevant endpoint CF, there was a significant association between high expression of miR-210 and worse CFFS for the mean cut-off (p = 0.001; Table 1 and Fig. 2b) and the trend towards worse CFFS was present for all cut-offs. The result was validated in the different cohorts by stratifying for Health authority regions (Northern Norway, n = 307, p = 0.020; Central Norway, n = 228, p = 0.024). The same trend was also significant for most clinical relevant subgroups (age, preoperative PSA, Gleason score, surgical margins, pT-stages, nerve infiltration, tumor-size and vascular infiltration).

### Multivariate analyses

In multivariate analyses for BFFS, neither the tumor cell nor the fibroblast miR-210 expression variable could be entered into multivariate analyses due to non-significance in univariate analyses. For CFFS, both the tumor cell and fibroblast miR-210 expression variable were entered in the multivariate analyses along with significant clinicopathological variables. Fibroblast miR-210 expression was independently associated to CFFS. Patients with a high fibroblast expression of miR-210 had a HR = 2.76 (CI 95% 1.25–6.09) compared to patients with a low expression (p = 0.012). See Table 2 for details regarding multivariate analysis. Tumor cell miR-210 expression was removed at an early stage in the backward conditional analysis due to non-significance when entered along with the other variables.

## Discussion

In our large PC cohort, we found fibroblast miR-210 expression, but not tumor cell expression to be independently associated to a poor CFFS. This prognostic impact was stronger than for any of the renowned clinicopathological features except Gleason score.

Hitherto, this is the second ISH study on miR-210 in any cancer and the first in prostate cancer. In addition to novelty, the major strength of this study is the large and well-described multicenter cohort with a long follow-up, enabling internal validation of the results between geographically different cohorts subjected to different tissue handling. The ISH technique is unique in its ability to evaluate the expression in a tissue specific context, which is of outmost importance in a multifocal tumor like PC. A potential drawback of ISH is potential low sensitivity, but for the abundant miR-210 expression found in this study, the ISH technique seems suited. The stringent use of mean cut-offs in the present study also makes the results easier to reproduce. The fact that stromal fibroblast were only morphologically identified, is a possible weaknesses. However, highly specific fibroblast markers are disputed and samples were scored by two experienced pathologists. Our tumor cell results, showing only a trend towards association with CFFS (p = 0.09), were possibly insignificant due to the large number of cores (35%) with no morphologically verified malignant cells. However, this fact only highlights the importance of *in situ* evaluation in PC.

No previous study has evaluated the prognostic impact of miR-210 in PC. Three different meta-analyses with systematic reviews of the prognostic impact of miR-210 in carcinomas were published recently, two in 2014^15^,^16^ and one in 2015^23^. All studies were based on qRT-PCR. Wang *et al*.^16^ included 16 studies incorporating 1809 patients spread on seven different cancer groups. Li *et al*.^15^ included nine studies with 1238 patients on five cancer groups and Xie *et al*.^23^ included 10 studies with a total of 777 patients on nine groups. Although the meta-analysis of Wang *et al*. did not include the later results from Lai *et al*.^24^ on 125 glioblastmas or from Qu *et al*.^25^ on 193 colorectal carcinomas, both showing overexpression of miR-210 associated with poor prognosis, this review seems to be the most comprehensive hitherto. Almost all the individual studies in this review showed statistically significant and independent associations between miR-210 overexpression and poor survival end-points. Exceptions were a bladder cancer^26^ and a metastatic breast cancer study^27^. Across all cancers in the metaanalysis, overexpression was only trending towards an association with poor OS (p = 0.21), but for the other survival end-points (DFS, PFS, RFS), overexpression was significantly associated with poor a prognosis. These studies point towards an association between miR-210 and poor prognosis, but conflicting results may be due to small cohorts or poorly controlled tissue sampling with a mixture of tumor and stromal cells. In prostate cancer, miR-210 is overexpressed in tumor tissue compared to controls^17^. However, the overexpression in the tumor might also be localised to the fibroblasts, not only the tumor cells. It should however be noted that the different associations to prognosis between cancer types might also point to a true difference in functional effect of miR-210 expression. Our previous ISH study with the same methodology in a large NSCLC cohort observed an association to a better prognosis with a high expression in both tumor and stroma^20^. Clinically these cancers are very different regarding phenotype and prognosis suggesting very different biological drivers in these cancers.

**Figure 3:**
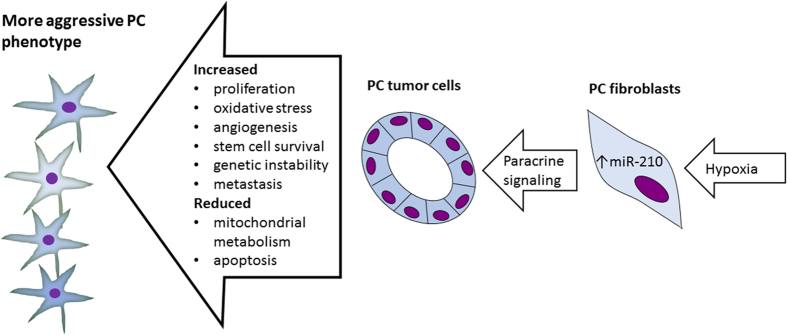
Under hypoxia, fibroblasts are induced to senescence and a cancer associated fibroblast (CAF) phenotype. These CAFs can secrete miR-210, which induces malignant transformation to the prostate cancer (PC) cells, yielding a more malignant phenotype mediating a poorer prognosis. Figure based on references^25^,^32^,^35^. Abbrevations: EMT = endothelial mesenchymal transformation, PC = Prostate cancer.

The role of miR-210 in cancer progression is studied more thoroughly than many other miRs due to its widely accepted role as a master regulator of hypoxia response and its aberrant expression in different carcinomas^28^ (see Fig. 3). miR-210 expression is induced by HIF1α^29^, HIF2α^30^ and NFκB^31^ upon hypoxia. It has a number of targets and thereby yield various effects usually associated with a hypoxic phenotype; increased stem cell survival, repression of mitochondrial metabolism, promotion of cell cycle progression, increase of genetic instability, evasion of apoptosis and promotion of angiogenesis and metastasis (reviewed in refs 19, 28). An additional study found cancer cell secretion of miR-210 to serve as an angiogenic miRNA in endothelial cells^32^ and the reversed relationship between stroma and tumor for miR-210 has also been proposed^33^. Corroborating these results, our study found a weak correlation between VEGFR-2 and miR-210 in fibroblasts, pointing towards association between known angiogenic markers and miR-210. On the other hand, there are also reports of miR-210 acting as a tumor suppressor^34^,^35^,^36^. A more novel finding was the association to the pan-T lymphocyte co-receptor CD3. This finding points to a possible connection to lymphatic cell infiltration, which in many cancers has gained increasing interest.

Incidence of PC is strongly associated to high age, and it is postulated that stromal senescent cells at advanced age may be metabolically active, secreting factors promoting carcinogenesis and tumor progression^37^,^38^. In an elegant study by Taddei *et al*.^22^ the role of senescent fibroblasts in onset and progression of PC was explored. They found miR-210 overexpression to convert young fibroblasts into cancer-associated fibroblasts (CAFs), able to induce endothelial mesenchymal transition (EMT), angiogenesis, aerobic glycolysis and immunological adaptations. This gives a functional rationale for the association between high expression of miR-210 in fibroblasts and poor clinical outcome in prostate cancer patients. We have previously highlighted the importance of the tumor-stroma interplay in PC through identification of a metabolic fibroblast phenotype utilizing aerobic glycolysis and tumor cells using lactate, to be associated to a poor prognosis^39^.

This study adds to the growing literature on the importance of the tumor microenvironment in general, and fibroblasts in particular. Fibroblasts are no longer regarded as supportive connective tissue only, but can act as active contributors to the malignant phenotype^40^. However, this knowledge might implicate some possible gains. A poor prognostic relevance of CAFs is postulated, but to conclusively define these “enemies” are difficult^41^. Identifying functional attributes of the fibroblasts might select those with the strongest impact on prognosis. Overexpression of miR-210 is such a candidate marker and can be analysed on routine paraffin-embedded tissue by ISH.

In addition, miR-210 presents itself as a possible therapeutic target. Although it is involved in extensive areas of cell function and might thereby be considered a non-specific (dirty) target, its expression levels are directly linked to the hypoxia metagene making it a function-specific target^12^. Anti-miR treatments can be antagomirs, miR-maskes, LNA probes, siRNAs, miR-sponges or other approaches that could target specific miRs^19^,^42^ but none are beyond clinical phase I trials (clinicaltrials.gov). Combining anti-miR-210 treatment and existing therapies (chemotherapy and radiotherapy) is a plausible approach due to the inherent resistance of hypoxic cells to these treatments. Yet, only one *in vitro* prostate cancer study has been published, but disappointingly without signs of effect of a miR-210 inhibitor when combined with radiotherapy^43^.

## Conclusions

This is the first study of *in situ* miR-210 expression in prostate cancer. Our approach adds to the current understanding of fibroblasts and their role in tumor progression. Our data reveal miR-210 as a validated independent negative prognostic marker in PC, and leads to the proposition that miR-210 is a potential therapeutic target in this multifocal malignancy.

## Materials and Methods

### Patients and tissue micro array

671 patients were retrospectively identified with radical prostatectomies (RPs) for adenocarcinoma of the prostate between 01.01.1995 to 31.12.2005 from the archives of the Departments of Pathology in two health regions in Norway. Of these, 131 patients were excluded, leaving 535 eligible patients with complete follow-up data and available tissues from St. Olav Hospital/Trondheim University Hospital (St. Olav) in the Central Norway region (n = 228), and Nordlandssykehuset Bodo (NLSH) (n = 59) and the University Hospital of North Norway (UNN), both in Northern Norway region (n = 248).

Biochemical failure (BF) was defined as a PSA ≥ 0.4 ng/ml and. BF-free survival (BFFS) was calculated as time from surgery to last follow up (FU) date or date with PSA above threshold. Clinical failure (CF) was defined as symptomatic, locally advanced progression or metastasis to bone, visceral organs or lymph nodes verified by radiology. Clinical failure-free survival (CFFS) was calculated from date of surgery to last FU date without CF or to date of CF. For more extensive information regarding patients, exclusion, definitions of variables and endpoints, see our previous report^21^. Last update was in December 2015. Median follow up of survivors was 150 months.

Twelve tissue micro array (TMA) blocks were constructed from this cohort as follows: We collected formalin-fixed paraffin-embedded (FFPE) tissue blocks from included patients. The pathologist (ER) sampled two areas of the most dedifferentiated neoplastic cell compartment (tumor) and two areas from adjacent tumor stroma (stroma). We used a tissue-arraying instrument (Beecher Instruments, Silver Springs, MD, USA) to harvest cores with a diameter of 0.6 mm from these areas. These cores were inserted into paraffin blocks. A Micron microtome (HM355S) was used to cut 4 μm sections. The sections were affixed to glass slides and finally sealed with paraffin.

### *In situ* hybridization

We optimized the “One–day microRNA ISH protocol” developed by Exiqon, Vedbek, Denmark for specific and sensitive detection of miRs by *in situ* hybridization in FFPE sections. Probes used in this study were digoxigenin (DIG) labelled locked nucleic acid (LNA) modified probes from Exiqon; for miR-210 (18103-15, hsa-miR-210), the positive control (99002-15, U6, hsa/mmu/rno) and the negative control (99004-15, scramble-miR).

We attached 4 μm sections from the TMA blocks to Super Frost Plus slides by over night heating at 59 °C These sections were further placed in xylene (3 × 10 min.) before hydration by adding ethanol solutions to PBS at pH 7.4. Treatment for Proteinase-K (Exiqon Kit 9000), 20 μg/ml, was done in 20 min using a PK-buffer (5 mM Tris.HCl, pH 7.5, 1 mM EDTA, 1 mM autoclaved NaCl) at 37 °C in a ThermoBrite hybridizer. Subsequent dehydration was achieved by adding increasing gradients of ethanol solutions before air-drying. Denaturation of the LNA-probes was achieved by heating to 90 °C for 4 min. Hybridization of the LNA-probes miR-210 (50 nM), scramble miR (50 nM) and U6 (2 nM) was done in a ThermoBrite hybridizer at 55 °C for 60 min. Thorough washing was done in pre-heated SSC buffers at 55 °C (VWR A 1396), 1 × 5 min. in 5× SSC, 2 × 5 min in 1× SSC and 0, 2× SSC. Unspecific binding was blocked by exposing sections to blocking solution for 15 min. at room temperature (RT). The blocking solution was the DIG wash and Block Buffer set (11 585 762 001, Roche, Mannheim, Germany). For immunologic detection, the alkaline phosphatase (AP)-conjugated anti—DIG (11 093 274 910 Roche) at 1:800 was used for incubation for 30 min. at RT. Following the PBS-T wash, we did the substrate enzymatic reaction with NBT/BCIP (11 697 471 001, Roche) at 30 °C in the ThermoBrite for 2 hours. To stop the reaction, we washed with a KTBT buffer (50 mM Tris-Hcl, 150 mM NaCl, 10 mM KCl) for 2 × 5 min. Counter staining of section was done by 1 min use of nuclear fast red (WALDECK, ZE-012-250) at RT before tap water rinse. The last steps were dehydration by ethanol at increasing gradients and mounting by use of the Histokitt mounting medium (Assistant-Histokitt, 1025/250). For staining controls please see Supplementary Fig. S1.

### Scoring of *in situ* hybridization and cut-off

In tumor cells we scored intensity for miR-210 semiquantatively as 0 = negative, 1 = weak, 2 = moderate and 3 = strong. Density (percentage) was not scorable in tumor cells due to homogenous staining when present. In fibroblasts, miR-210 intensity was scored as in tumor (0–3). Density in fibroblasts was scored as follows: 0 = not present; 1 = 1–24%, 2 = 25–75%; 3 > 75%. Two pathologists (L.M. and E.R.) independently reported the score for morphologically verified tumor cells and fibroblasts. They were blinded to each other’s score and the outcome of the patient. In fibroblasts, the semi-quantitative intensity- and density scores were summarised to determine the total expression level of miR-210 (0–6). Mean cut-offs for dichotomization were used for both tumor cell and fibroblast scores.

### Statistical analyses

Statistical analyses were done using the SPSS software version 22 (IBM SPSS Inc., Chicago, IL, USA). Inter-observer reliability of pathologist scoring was tested by use of a two-way random effect model with absolute agreement. Associations between miR-210, previously published molecular markers and clinicopathological markers were analysed using the the Spearman’s Correlation test. The Kaplan-Meier method was used for drawing plots of clinical failure-free survival and statistical significance of difference was assessed by use of the log-rank test. The curves were terminated when less than 10% of patients were still at risk (192 months). Variables from the univariate analyses with a p < 0.10 were assessed for significant and independent impact on CFFS in a stepwise backward multivariate Cox regression model with a probability at 0.05 for entry and 0.10 for removal. The chosen significance level for all analyses was p < 0.05.

### Ethics

This study was approved by the regional ethics committee, REK Nord, project application 2009/1393, including a mandatory re-application which was formally approved again 22.01.2016. The committee waived the need for patient consent for this retrospective study. The reporting of clinicopathological variables, survival data and biomarker expressions was conducted in accordance with the REMARK guidelines.

## Additional Information

**How to cite this article**: Andersen, S. *et al*. Fibroblast miR-210 overexpression is independently associated with clinical failure in Prostate Cancer – a multicenter (*in situ* hybridization) study. *Sci. Rep.* **6**, 36573; doi: 10.1038/srep36573 (2016).

**Publisher’s note:** Springer Nature remains neutral with regard to jurisdictional claims in published maps and institutional affiliations.

## Supplementary Material

Supplementary Information

## Figures and Tables

**Table 1:** atient characteristics and clinicopathological variables, and their prognostic value for the biochemical and clinical failure in 535 prostate cancer patients (univariate analyses; log rank test).

Characteristic	Patients (n)	Patients (%)	BF (200 events)		CF (56 events)	
			5-year EFS (%)	p	10-year EFS (%)	p
**Age**				0.237		**0.038**
≤ 65 years	357	67	77		94	
>65 years	178	33	70		91	
**pT-stage**				**<0.001**		**<0.001**
pT2	374	70	83		97	
pT3a	114	21	61		87	
pT3b	47	9	43		74	
**Preop PSA**				**<0.001**		**0.029**
PSA < 10	308	57	81		95	
PSA > 10	221	42	68		89	
Missing	6	1	—		—	
**Gleason**				**<0.001**		**<0.001**
3 + 3	183	34	83		98	
3 + 4	219	41	77		94	
4 + 3	81	15	70		90	
4 + 4	17	4	58		86	
≥9	35	6	37		65	
**Tumor Size**				**<0.001**		**0.002**
0–20 mm	250	47	83		96	
>20 mm	285	53	68		90	
**PNI**				**<0.001**		**<0.001**
No	401	75	80		96	
Yes	134	25	60		83	
**PSM**				**0.049**		0.198
No	249	47	81		96	
Yes	286	53	69		90	
**Non-apical PSM**				**<0.001**		**<0.001**
No	381	71	82		96	
Yes	154	29	57		85	
**Apical PSM**				0.063		0.427
No	325	61	74		92	
Yes	210	39	77		93	
**LVI**				**<0.001**		**<0.001**
No	492	92	77		95	
Yes	43	8	47		69	
**Surgical proc**				0.466		0.308
Retropubic	435	81	77		92	
Perineal	100	19	68		95	
**Tumor cell miR-210**				0.85		0.09
High expression	162	30	73		91	
Low expression	183	34	73		94	
Missing	190	36				
**Fibroblast miR-210**				0.23		**0.001**
High expression	310	58	74		90	
Low expression	187	35	79		97	
Missing	38	7				

Abbreviations: BF = biochemical failure; CF = Clinical failure; EFS = event free survival in months; LVI = lymphovascular infiltration, PCD = prostate cancer death; NR= not reached; P = P value for log rank statistic for difference in event free survival; PC = Prostate cancer; PNI = Perineural infiltration; Post op RT = postoperative radiotherapy; Preop = preoperative; PSA=Prostate specific antigen; PSM = Positive surgical margin; Surgical proc = surgical procedure;

**Table 2:**
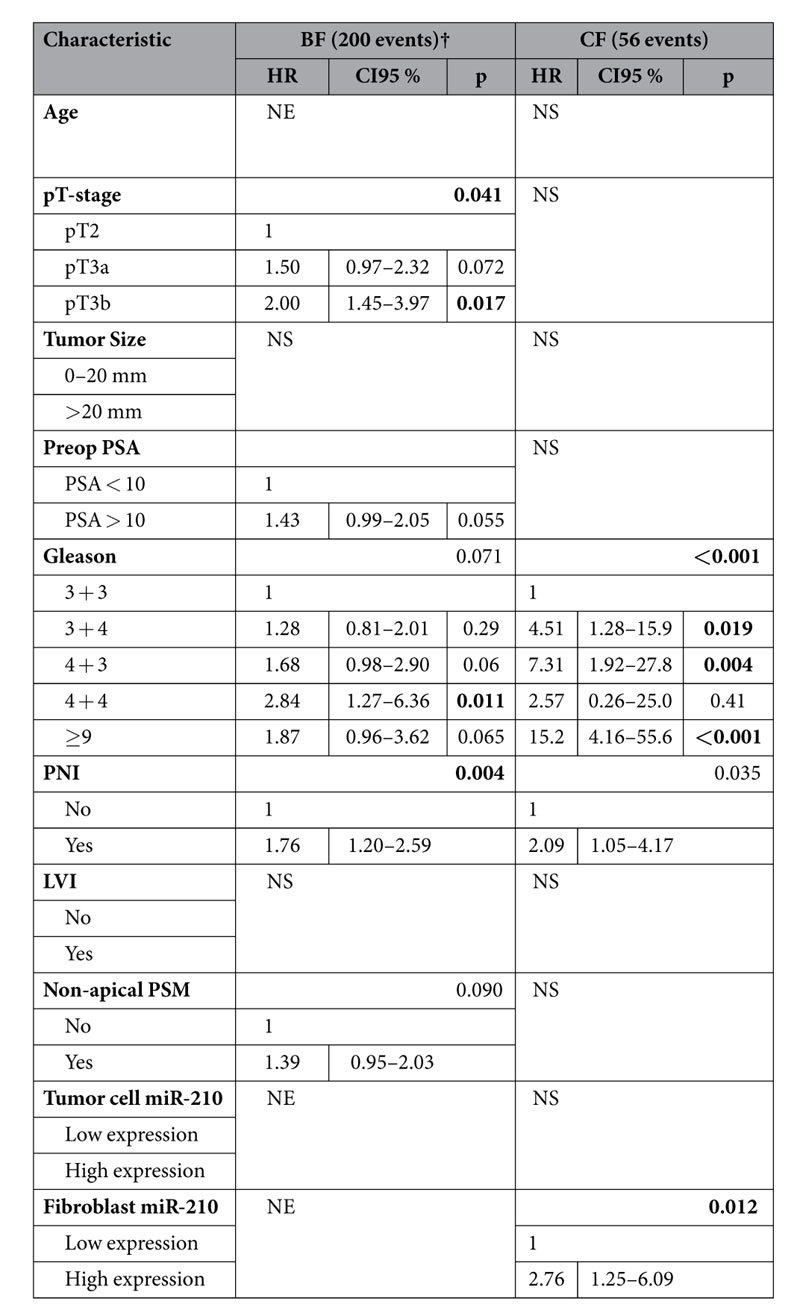
Multivariate analyses of factors with a p < 0.10 in univariate analyses (see Table 1) for all patients (Cox regression, backward conditional).

Significant p-values in bold (threshold p ≥ 0.05). Abbreviations: BF = biochemical failure; CF = Clinical failure; LVI = lymphovascular infiltration; NE = not entered, due to non-significance in the univariate analyses; NS = not significant, the characteristic is removed by the backward conditional analysis due to insignificance; PNI = Perineural infiltration; Preop = preoperative; PSA = Prostate specific antigen; PSM = Positive surgical margin.
