# Supplementary material for: Fibroblast miR-210 overexpression is independently associated with clinical failure in Prostate Cancer – a multicenter (in situ hybridization) study
Source: Sci Rep. 2016 Nov 8;6:36573. doi: 10.1038/srep36573 (PMC5099893; doi:10.1038/srep36573)
Supplement: Supplementary Information [file srep36573-s1.pdf]

Fibroblast miR-210 overexpression is independently associated with clinical failure in Prostate Cancer – a multicenter (*in situ* hybridization) study

Sigve Andersen, PhD, M.D[1,2]
Elin Richardsen, PhD, M.D[3,4]
Line Moi PhD, M.D[3,4]
Tom Donnem, PhD, M.D[1,2]
Yngve Nordby, M.D [1,5]
Nora Ness, [3]
Marte Eilertsen Holman, PhD, M.D[1,2]
Roy M. Bremnes, PhD, M.D[1,2]
Lill-Tove Busund, PhD, M.D[3,4]

[1]Translational Cancer Research Group, Dept Clinical Medicine, UiT, The Arctic University of Norway
[2]Dept Oncology, University Hospital of North Norway, Tromso
[3]Translational Cancer Research Group, Dept of Medical Biology, UiT, The Arctic University of Norway
[4]Dept Pathology, University Hospital of North Norway, Tromso
[5]Dept of Urology, University Hospital of North Norway, Tromso

Corresponding author:
Sigve Andersen, PhD, MD
Department of Oncology
University Hospital of North Norway
9038 Tromso, Norway
Telephone: +47 77 62 60 00 Fax: +47 77 62 67 79
E-mail: sigve.andersen@uit.no

Staining controls of miR-210

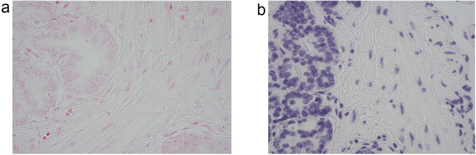

400x microscope pictures of a) negative control of miR-210 staining (scramble) and b) positive control of miR-210 staining (U6).
